# Assessing the Effect of Modified Clay on the Toxicity of *Karenia mikimotoi* Using Marine Medaka (*Oryzias melastigma*) as a Model Organism

**DOI:** 10.3390/toxics10030105

**Published:** 2022-02-23

**Authors:** Peipei Zhang, Xiuxian Song, Yue Zhang, Jianan Zhu, Huihui Shen, Zhiming Yu

**Affiliations:** 1 Key Laboratory of Marine Ecology and Environmental Sciences, Institute of Oceanology, Chinese Academy of Sciences, Qingdao 266000, China; zhangpeipei@qdio.ac.cn (P.Z.); yzhang4@qnlm.ac (Y.Z.); zhujn@qdio.ac.cn (J.Z.); shenyghui@163.com (H.S.); zyu@qdio.ac.cn (Z.Y.); 2 Laboratory of Marine Ecology and Environmental Science, Qingdao National Laboratory for Marine Science and Technology, Qingdao 266000, China; 3 University of Chinese Academy of Sciences, Beijing 100049, China; 4 Center for Ocean Mega-Science, Chinese Academy of Sciences, Qingdao 266000, China

**Keywords:** modified clay, *Karenia mikimotoi*, harmful algal bloom, marine medaka, marine toxicity tests

## Abstract

Blooms of the toxic dinoflagellate *Karenia mikimotoi* could threaten the survival of marine life, and modified clay (MC) is considered a promising method for the control of harmful algal blooms. Here, using marine medaka as the model organism, the toxicity of *K. mikimotoi* before and after MC disposal was investigated. The results showed that only a certain density of intact *K. mikimotoi* cells could cause obvious damage to fish gills and lead to rapid death. A systematic analysis of morphology, physiology, and molecular biology parameters revealed that the fish gills exhibited structural damage, oxidative damage, osmotic regulation impairment, immune response activation, and signal transduction enhancement. MC can flocculate *K. mikimotoi* rapidly in water and reduce its toxicity by reducing the density of intact algae cells and hemolytic toxicity. The results indicate that MC is an effective and safe method for controlling *K. mikimotoi* blooms.

## 1. Introduction

*Karenia mikimotoi* is a toxic species of harmful algal blooms (HABs), and the species form HABs in the offshore waters of many countries or regions, causing many marine organisms to die and the marine aquaculture and fisheries to incur huge economic losses [1]. For example, *K. mikimotoi* blooms were recorded in the northern half of the west coast of Ireland (2005), Scottish waters (2006), and Hakodate Bay in northern Japan (2015). At the same time, it has been widely reported that large numbers of echinoderms, annelids, molluscs and fish have died [2,3,4]. In 2012, the HABs of *K. mikimotoi* in the coastal area of Fujian, China, caused many abalone *(Haliotis discus hannai)* and fish to die, causing economic losses of more than USD 330 million [5]. As *K. mikimotoi* blooms occur frequently and cause extensive harm, they have become a widespread concern in society.

At present, the cause and mechanism of *K. mikimotoi*-induced death are still unclear, and scientists have proposed many related hypotheses. It is believed that *K. mikimotoi* may cause a hypoxic environment, produce lipophilic toxins, cause oxidative damage and other effects, leading to many marine deaths [6,7]. The composition of toxins in *K. mikimotoi* is not clear, and *K. mikimotoi* does not produce common algae toxins, such as brevetoxin (BTX), paralytic shellfish toxin (PSP), diarrhea shellfish toxin (DSP), or karlotoxin [1,8,9,10]. Currently, the known toxic components of *K. mikimotoi* include hemolytic toxins [11,12], gymnocin-A, gymnocin-B [13,14], and polyunsaturated fatty acids (PUFAs) [15]. The toxicity of these substances extracted in the laboratory is usually lower than that of intact algae cells [14]. Studies showed that the intact cell of *K. mikimotoi,* rather than the cell-free culture supernatant and the ruptured cell suspension, had the most toxic effects on marine organisms. *K. mikimotoi* from Fujian coastal waters significantly affected the tested organisms, including *Brachionus plicatilis*, *Artemia salina*, *Calanus sinicus*, *Neomysis awatschensis*, *Penaeus vannamei* and *Scophthalmus maximus,* which had mortality rates at 96 h of 100, 23, 20, 97, 33, and 53%, respectively, whereas cell-free culture and the ruptured cell suspension had no significant effects on the tested organisms [5]. After 10 h incubations, the survivorship of rotifers *B. plicatilis* exposed to *K. mikimotoi* (SUO-1) was 20%, and both the cell-free culture supernatant and the ruptured cell suspension were not toxic to rotifers [12]. Both field and laboratory studies have shown that, compared to other organisms, fish were more sensitive to *K. mikimotoi*. In many cases of *K. mikimotoi* blooms, only fish were recorded to be poisoned, while other biological groups were less affected.

Marine medaka (*Oryzias melastigma*) is a model organism widely used in marine and estuarine ecotoxicology studies. It has advantages including a small size, short generation cycle, distinct sexual dimorphism, simple culture and breeding process, and sensitivity to environmental pollutants [16,17,18]. The adult fish affected by *K. mikimotoi* blooms are generally larger and less sensitive to pollutants than marine medaka. The external stimuli that marine medaka can withstand represent the situation of many commercial fish adults and juveniles to some extent. Gills are organs involved in gas exchange, osmotic adjustment, and acid-base adjustment and are the most direct organs that contact toxic substances in water [19]. *K. mikimotoi* can damage gills in various manners, such as epithelial cell shedding or atrophy, tissue adhesion, and inhibition of chloride cell functions. At the same time, excessive mucus production and tangled filaments on the surface of fish gills can be observed. This damage may disrupt the normal functions, such as respiration and osmotic regulation of the fish gills, which may cause the fish to suffocate and die [2,20,21,22]. Therefore, the gills of marine medaka are ideal materials for studying the toxicity of *K. mikimotoi*.

Modified clay (MC) has been successfully used for on-site treatment of HABs in China. MC technology has been recommended by UNESCO and APEC [23] and included as a national standard method (GB/T 30743-2014) in China [24]. MC can effectively remove various HAB species, such as *K. mikimotoi*, *Alexandrium pacificum*, and *Aureococcus anophagefferens* [25,26,27]. At the appropriate dosage different kind of clay or MC does not have harmful effects on fish (such as *Scophthalmus maximus* L. embryos [28], *Pagrus major* and *Paralichthys olivaceus* [29], *Chanos chanos* Forsskal, *Lates calcalifer* Bloch *and Siganus guttatus* Bloch [30], *Salmo salar* [31]), abalone *Haliotis discus hannai* [32], juvenile *Apostichopus japonicus* Selenka [33], and *Litopenaeus vannamei* [24]. Filter-feeding bivalve species are susceptible to sedimentation flocs; therefore, clay or MC will have certain adverse effects on some sensitive shellfish. *Placopecten magellanicus* had poor tolerance to suspended bentonite [34]. Yellow loess had different effects on the clearance rates of different bivalve species [35]. For example, the clearance rate of *Argopecten irradians* decreased with 0.01 g/L yellow clay treatment, while the clearance rate of *Crepidula fornicate* was not affected by yellow clay at concentrations less than 10 g/L. Many studies have shown that while clay or MC removed toxic microalgae, it also had a good ability to remove algae toxins, such as brevetoxin produced by *K. brevis* [36], *Prymnesium parvum* and its toxins [37], microcystin produced by *Microcystis*, *Anabaena* and *Oscillatoria* species [38], etc., in the water column. However, the effect of MC on the toxicity of *K. mikimotoi* is still unclear.

In this study, marine medakawas used as a model organism to compare the effects of *K. mikimotoi* on the survival and behaviour of the fish before and after MC flocculation. The toxic effects and mechanisms of *K. mikimotoi* on marine medaka were studied at different levels of external gill morphology, physiology, biochemistry, and gene transcription expression. The effect of MC on the toxicity of *K. mikimotoi* was comprehensively discussed to provide a theoretical basis for controlling *K. mikimotoi* blooms on-site with MC.

## 2. Materials and Methods

### 2.1. Organisms Culture and MC Preparation

In this study, *K. mikimotoi* isolated from waters near Fujian province, China, was provided by the Key Laboratory of Marine Ecology and Environmental Science, Institute of Oceanology, Chinese Academy of Sciences. Seawater was filtered through a 0.45 μm mixed fibre membrane and heated at 121 °C for 30 min, and L1 medium was then added for algal culture. The incubation temperature was 19 ± 1 °C, the light intensity was 70 μmol photons/(m^2^·s), and the light: dark cycle was 12 h:12 h.

The adult marine medaka used in the experiment were cultured in 0.45 μm mixed fibre membrane filtered seawater. The initial pH of the seawater was 7.9, the salinity was 32, the water temperature was maintained at 26 ± 1 °C, and the constant photoperiod was 12 h light:12 h dark. Through performing continuous aeration, the dissolved oxygen in the water was maintained at a level greater than 5 mg/L. Marine medaka were fed with *A. salina* twice a day. One week before the experiment, the culture temperature was slowly reduced to 20 °C by adjusting the heating rod until the experiment was carried out.

The MC used in the experiment was based on kaolin (Beihai, Guangxi Province, China) and was modified according to the method of Yu et al. [39]. The modifier was polyaluminium chloride (PAC, Guangfu Chemical Industry, Tianjin, China), and the modification ratio of PAC to kaolin was 1:5 (*w*/*w*). A suspension containing 25 g/L MC was prepared with seawater, which was used in the *K. mikimotoi* removal experiment.

### 2.2. Experimental Design

The microalgae density was counted daily using a counting chamber and a microscope (Olympus IX71, Tokyo, Japan). The experiment was carried out when the density of *K. mikimotoi* reached 3 × 10^4^ cells/mL. At this time, *K. mikimotoi* was in the late middle period of exponential growth.

The concentration of MC used in this experiment should meet the requirement of high removal rate of *K. mikimotoi* and no significant effect on the survival of marine medaka. 0.3 g/L of MC can remove 80% of *K. mikimotoi* cells in the water column, and it is lower than the safe concentration (1.94 g/L) of MC for newly hatched larvae of marine medaka [40]. Therefore, 0.3 g/L was chosen as the concentration of MC used in the experiment. 

#### 2.2.1. Effect of *K. mikimotoi* on the Marine Medaka

The experiment used a seawater filtered by 0.45 μm mixed fibre membrane to dilute *K. mikimotoi* suspension to a density of 1 × 10^3^, 3 × 10^3^, 5 × 10^3^, and 1 × 10^4^ cells/mL. *K. mikimotoi* suspension at a cell density of 3 × 10^3^ cells/mL was filtered to obtain the cell-free culture supernatant, and the cells were ruptured with an ultrasonic cell disruptor (JY92-11DN, Xinzhi, Ningbo, China) to obtain the ruptured cell suspension. The ultrasonic power was 200 W, the ultrasonic time was 5 s, the interval was 6 s, and the working time was 20 min. A little amount of ruptured cell suspension was taken and observed under the microscope. If there were no intact cells, the next step could be carried out. Seawater was used as the control group, and three replicates were used in each group. Five litres of the corresponding liquid were added to each container, and 10 fish were randomly placed to observe survival.

#### 2.2.2. Determination of Hemolytic Toxicity of *K. mikimotoi*

The algae cells were collected by filtration with GF/F membranes, and then membranes were extracted using a mixed solution of chloroform, methanol, and water with a volume ratio of 13:7:5. After nitrogen blowing, the solution was reconstituted with 1 mL methanol to obtain a crude extract, and then the crude extract was used to dissolve rabbit blood cells for experiments.

Took digitonin stock solution (10 μg/mL) 0, 0.10 mL, 0.15 mL, 0.20 mL, 0.25 mL, 0.30 mL, 0.35 mL, and added them to the centrifuge tube in turn. Took 0.10 mL of Triton X-100 and added it to the eighth centrifuge tube. 0.40 mL, 0.30 mL, 0.25 mL, 0.20 mL, 0.15 mL, 0.10 mL, 0.05 mL, and 0.30 mL of citrate buffer were added in sequence. Among them, a centrifuge tube with 0.40 mL of citric acid buffer was used as a negative control, and a centrifuge tube with Triton X-100 was added as a positive control. The 1.6mL shaken 0.5% rabbit red blood cell solution was added to the above centrifuge tube in turn. After heating in a water bath at 37 °C for 30 min, centrifuge at 800r/min for 10min, took the supernatant and detected the absorbance value A_i_ at a wavelength of 540nm, the absorbance value of the negative control is A_0_, the absorbance value of the positive control is A_c_, and calculate the hemolysis percentage (P_i_) of the working curve (Figure S1) according to the following formula.

$$\mathrm{hemolysis}\;\mathrm{percentage}\left(P_{i}\right)\left(\%\right)=\frac{\left(A_{i}-A_{0}\right)}{A_{c}}\times 100\%$$

During sample determination, blank control 1 was added with 0.10 mL methanol, 0.30 mL citric acid buffer, 1.6 mL rabbit red blood cell solution, the absorbance was A_b0_, and blank control 2 was added with 0.10 m of toxin crude extract and 1.9 mL citric acid buffer, and the absorbance was A_bw_. The sample was measured by adding 0.10 mL of toxin crude extract, 0.30 mL of citric acid buffer, 1.6 mL of rabbit red blood cell solution, the absorbance was A_w_, and the hemolysis percentage (Pa) and hemolysis activity (HA) was calculated according to the following formula.

$$\mathrm{hemolysis}\;\mathrm{percentage}\left(\mathrm{Pa}\right)\left(\%\right)=\frac{\left(A_{w}-A_{b0}-A_{\mathrm{bw}}\right)}{A_{c}}\times 100\%$$

$$\mathrm{hemolysis}\;\mathrm{activity}\left(\mathrm{HA}\right)=\frac{m}{{\mathrm{ED}}_{50}}\times 2\times 10\times \frac{1}{V}$$

where m is calculated according to the working curve, ED_50_ is the amount of digitalis saponins when P_i_ = 50%, and V is volume (L).

#### 2.2.3. Effect of MC on the Toxicity of *K. mikimotoi*

The fish were randomly divided into four groups (SW group, MC group, KM group, and the KMMC group), and three replicates were performed for each group. The experimental container was a 20 L plastic storage box. In the SW group, 16 L of seawater filtered with a 0.45 μm mixed fibre membrane was added. The MC group was sprayed with MC based on the SW group so that the final concentration of MC was 0.3 g/L. The density of the algal suspension that was decreased to 3 × 10^3^ cells/mL with filter seawater, and 16 L of the diluted algae liquid was added to the KM group. MC of 0.3 g/L was used in the KMMC group to remove *K. mikimotoi* with an algae density of 3 × 10^3^ cells/mL. Three hours after adding MC, 5 mL algae solution was fixed with Lugol’s solution, and then the algae cell density was counted under a microscope. The removal efficiency (RE) of MC on *K. mikimotoi* was calculated. After counting algae cell density, 30 marine medaka (female: male = 1:1) were put into each group, which was the initial time of each experimental group. A large number of marine medaka deaths occurred approximately 3 h after exposure to *K. mikimotoi*. Within a few minutes before death, the fish had a slow response and rollover. At this time, different parameters of each group were sampled. The experiment was carried out for 24 h. During the experiment, no food was fed, no water was changed, and the dissolved oxygen level in the seawater was maintained by aeration on the seawater surface.

The RE is calculated as follows:

$$\mathrm{RE}\left(\%\right)=\left(1-\frac{\mathrm{The}\;\mathrm{cell}\;\mathrm{density}\;\mathrm{of}\;\mathrm{KMMC}\;\mathrm{group}}{\mathrm{The}\;\mathrm{cell}\;\mathrm{density}\;\mathrm{of}\;\mathrm{KM}\;\mathrm{group}}\right)\times 100\%$$

### 2.3. Histological Morphology Examination

After exposure for 3 h, the fish were anaesthetized in an ice water bath, the surface water was removed with filter paper, and the tissues were quickly dissected. After rinsing with physiological saline, the gills were put into precooled 2.5% glutaraldehyde for fixation, and this was followed by washing, ethanol gradient dehydration, displacement, critical point drying, sample fixation with conductive adhesive, gold spraying, etc. Scanning electron microscopy was used to observe the gill surface structure of fish.

### 2.4. Measurement of the Biochemical and Physiological Indexes

Many pollutants may produce toxicity related to oxidative stress, and MDA is commonly used as parameter reflecting lipid peroxidation. The SOD-CAT system is the first line of defence against oxidative stress [41]. In each parallel group, the gills of six fish (male: female = 1:1) were dissected and frozen in liquid nitrogen and then stored at −80 °C. When determining parameters such as antioxidant enzymes, the gills were processed into a 10% tissue homogenate and centrifuged at 3000× *g* for 15 min to collect the supernatant. Superoxide dismutase (SOD) activity, catalase (CAT) activity, malondialdehyde (MDA) content and Na^+^/K^+^-ATPase (NKA) activity were determined through the corresponding kits of the Nanjing Jiancheng Bioengineering Institute. The protein concentration was determined by a Solarbio Bradford kit.

### 2.5. RNA Extraction, Library Construction and RNA Sequencing (RNA-Seq)

For each parallel group, the gills of six fish (male: female = 1:1) were dissected, frozen in liquid nitrogen, and then stored at −80 °C. RNA was extracted using TRIzol reagent (Invitrogen, Carlsbad, CA, USA), and RNA degradation and contamination was monitored on a 1% agarose gel. A NanoDrop2000 spectrophotometer (Thermo Scientific, Waltham, MA, USA) was used to detect the RNA concentration. An RNA Nano 6000 detection kit (Agilent Bioanalyzer 2100 system, Agilent Technologies, Santa Clara, CA, USA) was used to evaluate the RNA integrity.

After the extracted RNA samples were qualified, a sequencing library was constructed. Oligo magnetic beads (dT) were used for the enrichment of mRNA, and fragmentation buffer was added to randomly interrupt the mRNA. Using mRNA as a template, the first cDNA strand was synthesized with a six-base random primer (random hexamers), and buffer, dNTPs, RNase H and DNA polymerase I were then added to synthesize the second cDNA strand. AMPure XP beads were used to purify the cDNA. The purified double-stranded cDNA was then repaired, and an A tail was added. The sequencing adapter was connected, AMPure XP beads were used for fragment size selection, and the cDNA library was enriched by PCR. After the library was constructed, the qPCR method was used to accurately quantify the effective concentration of the library (effective concentration of the library > 2 nM) to ensure the quality of the library. After the qualified of the library was confirmed, sequencing was performed using the Illumina HiSeq™ 2500 platform.

### 2.6. Functional Annotation of Genes

By removing reads containing linkers and removing low-quality reads (including reads with an N ratio greater than 10%) and removing reads with a quality value Q ≤ 10 (accounts for more than 50% of the entire reads), high-quality clean data were obtained, and the downstream analysis was performed. Differentially expressed genes (DEGs) were identified using the GO database (http://geneontology.org (accessed on 15 February 2021)) and KEGG database (http://www.genome.jp/kegg/ (accessed on 15 February 2021)) for GO functional enrichment and KEGG pathway analysis and annotation.

### 2.7. Quantitative Real-Time PCR

To verify the accuracy of the RNA-seq data, 13 DEGs were detected by qRT–PCR. The specific primers for these genes are shown in Table S1. The expression levels of the candidate genes were evaluated using the 2^−ΔΔCt^ method [42] with the expression of β-2-microglobulin (b2m) as a reference standard.

### 2.8. Statistical Analysis

All experimental data in this experiment were expressed as the mean ± standard error. All data were analyzed by SPSS 22.0 software for one-way ANOVA with Tukey HSD. The confidence level *p* < 0.05 was significantly different, and the confidence level of *p* < 0.01 was very significantly different.

## 3. Results

### 3.1. Effects of MC on K. mikimotoi

#### 3.1.1. Effects of MC on *K. mikimotoi* Cell Morphology

**Figure 1:**
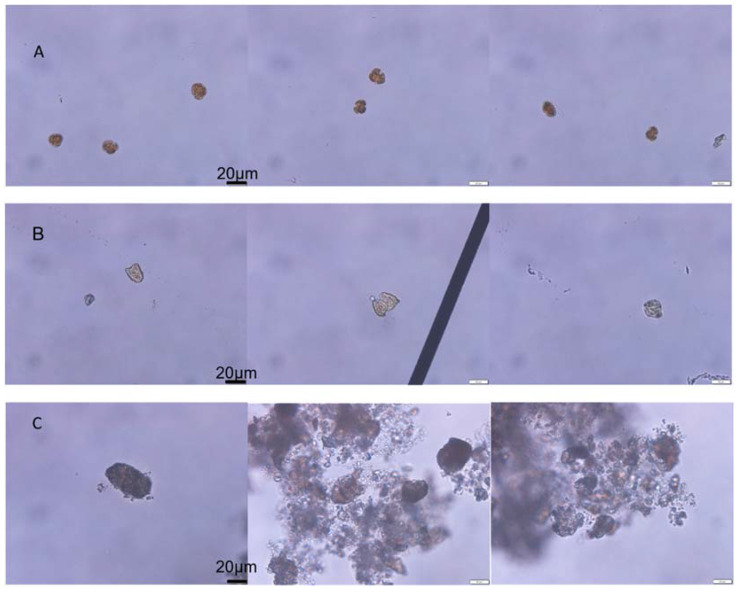
The effect of MC on the cell morphology of *K. mikimotoi.* (**A**) Morphology of the normal algal cell. (**B**) Morphology of the residual algal cells exposed by MC. (**C**) Morphology of the algae cells flocculated exposed by MC.

The initial algae cell density of *K. mikimotoi* was 3367 ± 225 cells/mL, and the RE of *K. mikimotoi* by MC was 83.67%. The algal morphology of the KM group and KMMC group is shown in Figure 1. The *K. mikimotoi* cells in the KM group had a complete cell morphology with clear cell edges. After adding MC, some of the residual algal cells in the water column had irregular shapes and blurry cell edges. Some cells were ruptured and no longer had a complete cell shape. The edges of the algae cells flocculated by the clay were blurred, the cytoplasm was eluted, and the algae cells died.

#### 3.1.2. Effect of MC on the Hemolytic Toxicity of *K. mikimotoi*

**Figure 2:**
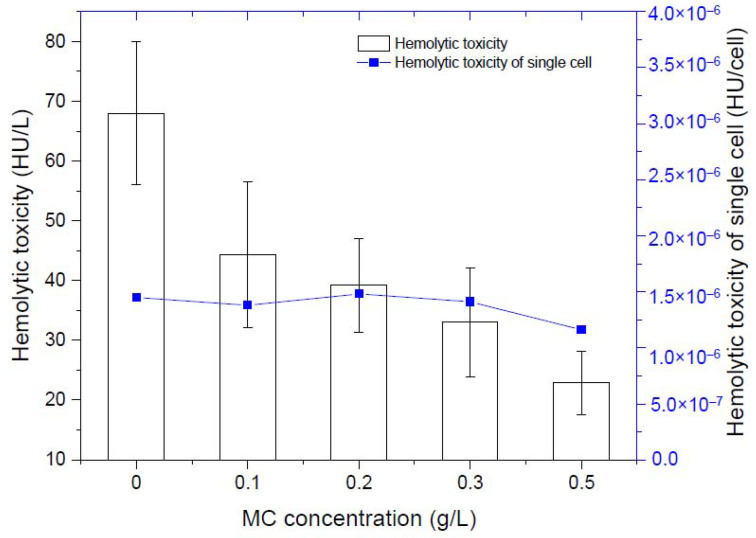
Changes of hemolytic toxicity of residual algal cells.

By measuring the hemolytic toxicity of *K. mikimotoi* at different densities, we found that the hemolytic toxicity of *K. mikimotoi* was significantly positively correlated with the algae cell density (Table 1). The higher the density the algae cells were, the stronger the hemolytic toxicity. There was no significant difference in the hemolytic toxicity of single residual algal cells before and after addition of MC, that is, MC less than 0.5 g/L did not stimulate the increased toxicity of residual algal cells. Due to the flocculation of MC, the number of intact algae cells in the water column was greatly reduced, so the total toxicity was significantly reduced (Figure 2).

### 3.2. Marine Medaka Survival

This study found that the toxicity of *K. mikimotoi* is directly related to the density of algae cells. When the density of algae cells is large enough, it will cause marine medaka to die. Its toxicity is also intact cell-dependent, and neither the cell-free culture supernatant nor the ruptured cell suspension has a lethal effect on marine medaka (Table 2). The marine medaka in the KM group died rapidly within 3 h, and the marine medaka had abnormal behaviours such as being unresponsive, rolling over, and floating on the water before death. All fish survived within 24 h in the SW group, MC group, and KMMC group, and there was no abnormal behavior.

### 3.3. Fish Gill Surface Morphology

**Figure 3:**
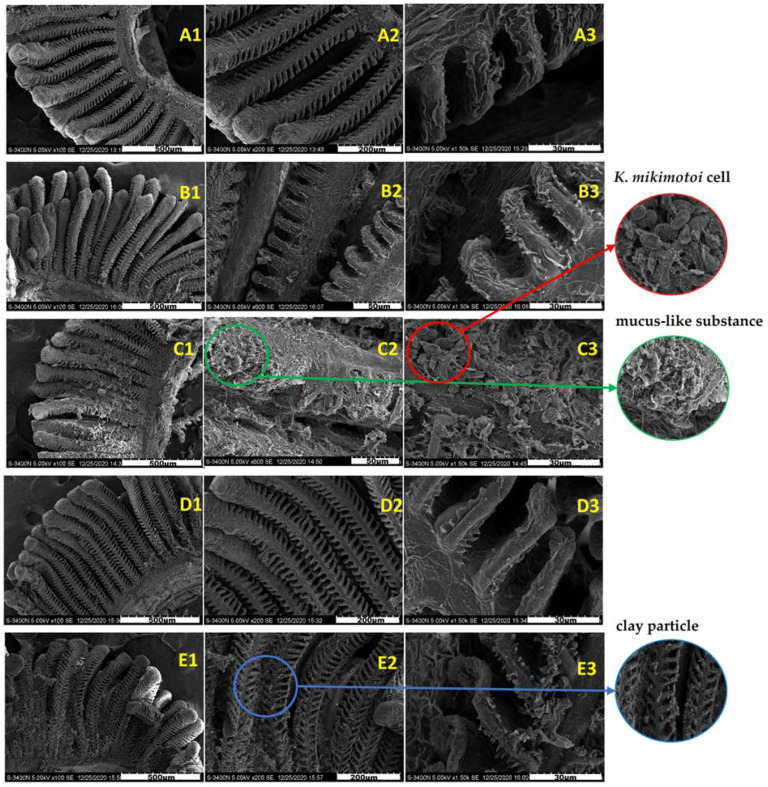
Surface morphology of the gills of different groups of marine medaka. (**A1**–**A3**) Surface. morphology of the normal gills in SW group. (**B1**–**B3**) Surface morphology of the gills exposed by MC. (**C1**–**C3**) Surface morphology of the gills exposed to *K. mikimotoi*. (**D1**–**D3**) Surface morphology of gills exposed in the KMMC group for 3 h. (**E1**–**E3**) Surface morphology of the gills exposed to KMMC for 24 h.

Figure 3 shows the surface morphology of the gills in different groups. The surface of the normal gill filaments was fluffy, and the gill lamellaes were neatly arranged and stable without adhesion (Figure 3A1–A3). Compared with the gills of the SW group, the gills of the KM group adhered to many algae cells, and a large amount of mucus was observed on the gill surface, resulting in the destruction of gill surface structure. However, there was no significant difference between the gills exposed to the KMMC group for 3 h and 24 h and the SW group. A small amount of algae cells and MC particles were attached to the surface of the gills. The magnified observation revealed that these particles did not damage the gills and that the gill structure was still intact. Compared with the SW group, the surface morphology of fish gills in the MC group did not change significantly.

### 3.4. Changes in the Gill Biochemical and Physiological Indexes

**Figure 4:**
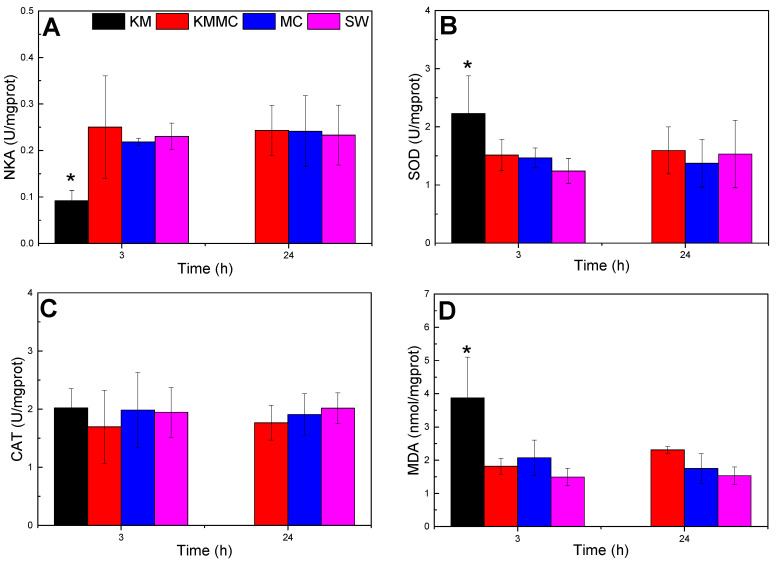
Changes in the biochemical and physiological indexes of different groups of marine medaka. (**A**) NKA activity, (**B**) SOD activity, (**C**) CAT activity, and (**D**) MDA content; * significant difference compared with SW group at *p* < 0.05.

As shown in Figure 4, compared with SW group, the NKA activity of the gills in KM group decreased significantly at 3 h (*p* < 0.05), the MDA content and SOD activity were significantly increased (*p* < 0.05), and the CAT activity exhibited no significant change (*p* > 0.05). The increase in MDA content indicated oxidative damage to gills, and the increase in SOD activity was a cellular response to oxidative stress. Compared with the SW group, there were no significant differences in NKA activity, CAT activity, SOD activity or MDA content at 3 h and 24 h in the KMMC group and MC group (*p* > 0.05). It indicated that the MC itself did not cause obvious oxidative stress to marine medaka, while the addition of MC significantly reduced the oxidative damage caused by *K. mikimotoi* to marine medaka.

### 3.5. Gene Expression

**Figure 5:**
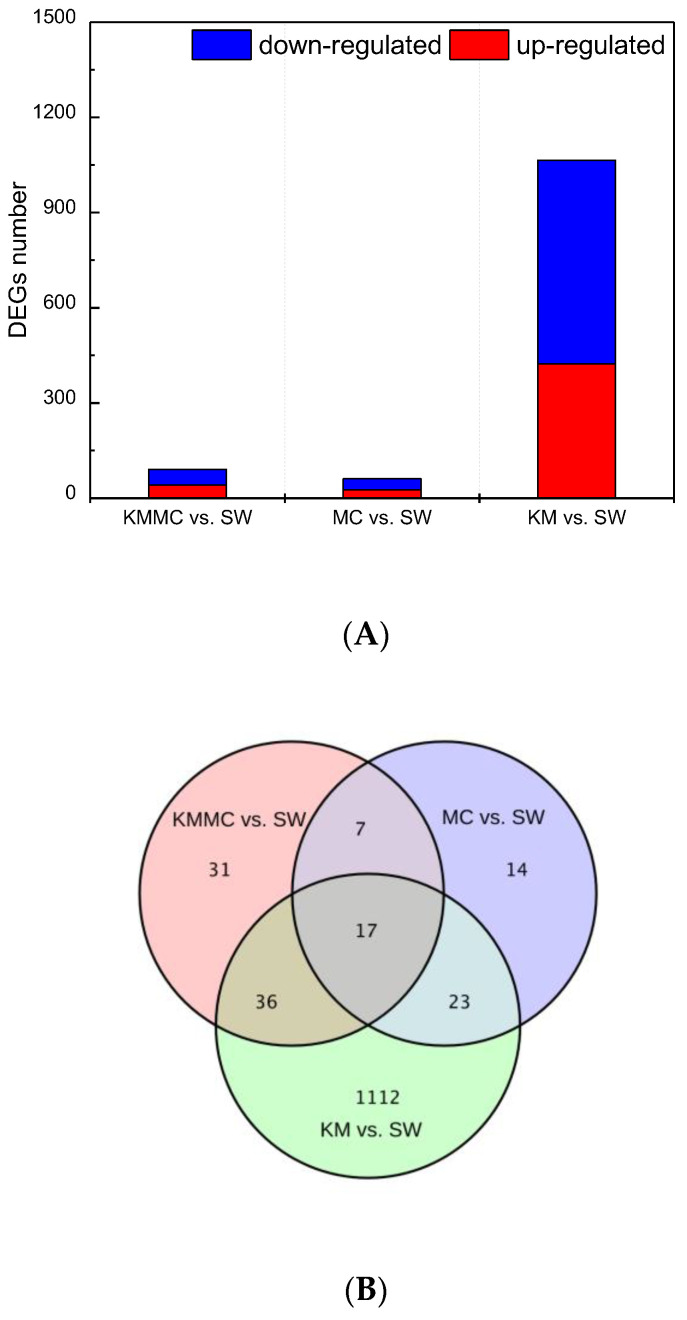
The number of DEGs in fish gills in each treatment group. (**A**) The numbers of up-regulated genes and down-regulated genes in each treatment group. (**B**) Venn diagram.

The DEGs between the KM group, MC group, KMMC group and SW group were screened by |log_2_ fold change| ≥ 1 and *p* < 0.05. Exposure to *K. mikimotoi* resulted in differential expression of many genes in fish gills (Figure 5A). In total, 452 genes were significantly up-regulated and 736 genes were significantly down-regulated (KM vs. SW). After removing *K. mikimotoi* with MC, the number of DEGs was significantly reduced. The numbers of significantly up-regulated genes and significantly down-regulated genes were reduced to 41 and 50, respectively (KMMC vs. SW). The number of DEGs in the MC group was even lower, and the numbers of significantly up-regulated genes and significantly down-regulated genes were 26 and 35, respectively (MC vs. SW). We further studied whether the different treatments affected the same set of genes and found that the number of DEGs common between the three treatments was only 17. For example, compared with the SW group, the KM group had 1112 genes that were unique (Figure 5B).

**Figure 6:**
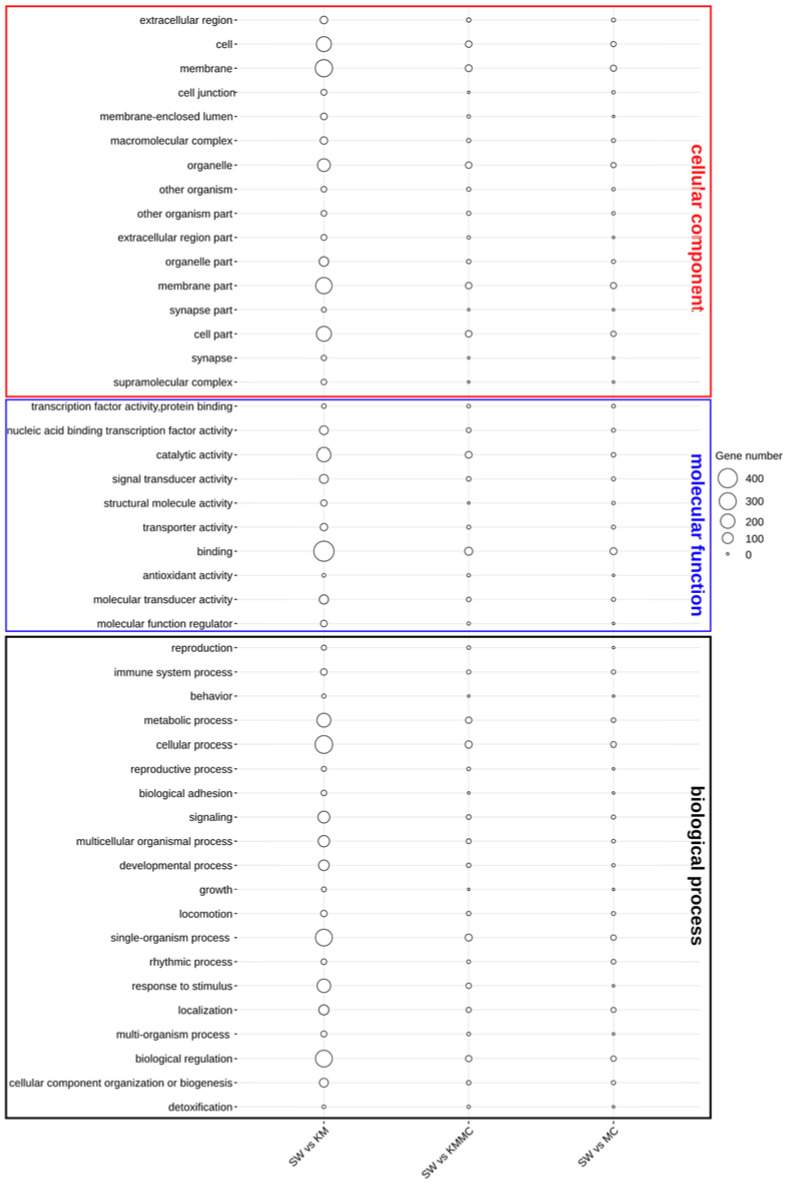
Catalogues of DEGs in biological process, cellular component, and molecular function items.

The functional annotation of DEGs was first characterized by the enrichment in GO terms at the biological process, cellular component, and molecular function levels (Figure 6). In the KM vs. SW and KMMC vs. SW groups, the DEGs in the biological process category were mainly related to signalling, response to stimulus, immune system process, organization process, cellular process, and metabolic process. The most important enriched GO terms in molecular function were the binding and catalytic activity, and the DEGs were significantly enriched in GO terms of cellular component in membrane and membrane part. Compared with KM vs. SW, the number of DEGs in KMMC vs. SW related to these terms in gills was significantly reduced.

**Figure 7:**
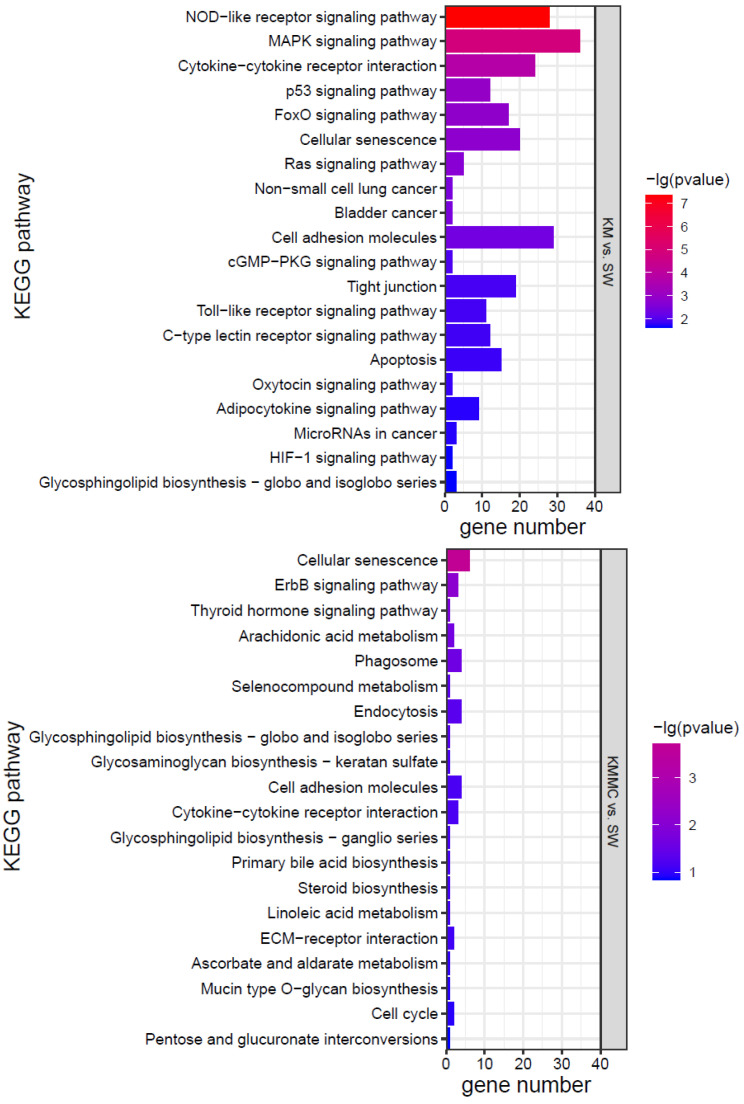

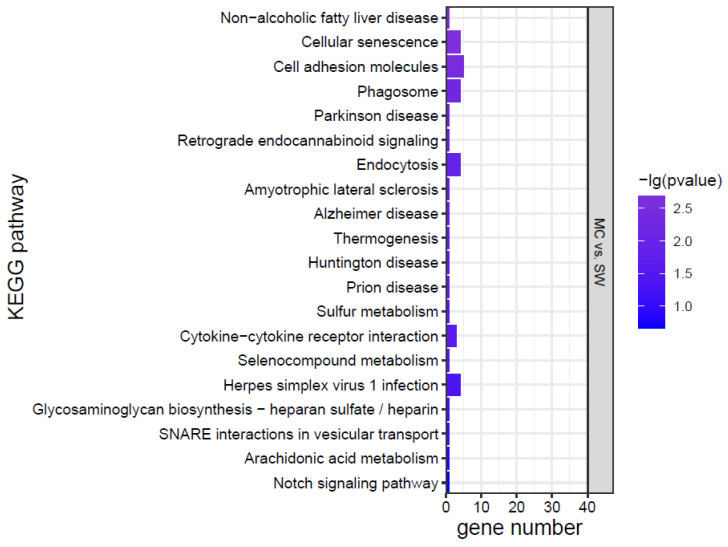
KEGG pathways of marine medaka gills in different treatment groups, *p* < 0.05 is significant.

KEGG analysis was conducted for the DEGs, and the top 20 KEGG pathways were screened according to the *p* value (Figure 7). The effects of *K. mikimotoi* on KEGG pathways in gills mainly focused on inflammation and immune responses (such as the NOD-like receptor signalling pathway, cytokine–cytokine receptor interaction, cell adhesion molecules, tight junctions, Toll-like receptor signalling pathway, and C-type lectin receptor signalling pathway), apoptosis and oxidative stress (such as the p53 signalling pathway and FoxO signalling pathway), substance metabolism, etc. After adding MC, the numbers of significantly enriched pathways and DEGs of each pathway were greatly reduced.

### 3.6. Data Validation by qRT-PCR

**Figure 8:**
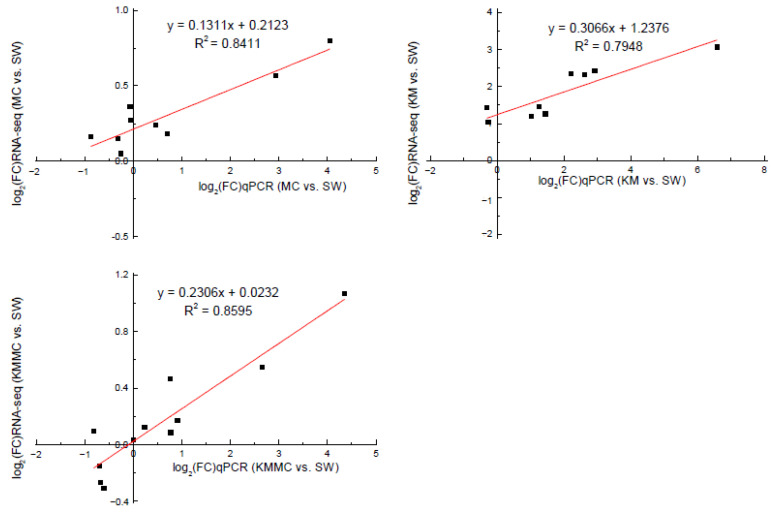
Correlation analysis between RNA-Seq and qRT-PCR validation results of genes.

In order to verify the accuracy of the RNA-Seq data, we selected 13 DEGs for qRT-PCR analysis. Correlation analysis showed that the gene transcript levels determined by these two methods had a relatively high linear correlation, indicating that the transcriptome sequencing data were reliable (Figure 8, Table S2).

## 4. Discussion

### 4.1. Marine Medaka Survival

As a method of treating HABs on site, MC is widely utilized for its ecological safety in China. Predecessors have performed much research on the safety of MC. The appropriate concentration of MC had no significant effect on the survival and growth of *Crassostrea gigas* juveniles, *Apostichopus japonicas Selenka* juveniles, *Patinopecten yessoensis* juveniles, *Mercenaria mercenaria*, *Haliotis discus hannai*, *Scophthalmus maximus* embryos, *O. melastigma* newly hatched larvae, etc [7,28,33,40,43,44,45]. The results of this study showed that 0.3 g/L MC did not have a significant impact on the survival of marine medaka.

Many previous studies have shown that intact *K. mikimotoi cells*, but not algae-free cell cultures or ruptured cell suspensions, were obviously toxic to *C. gigas* embryos, *A. nauplii*, *B. plicatilis*, *A. salina*, *C. sinicus*, *N. awatschensis*, etc [5,46]. Research by Zou et al. showed that if *K. mikimotoi* and *B. plicatilis* were separated by a semipermeable membrane, the survival rate of rotifers was not affected by *K. mikimotoi* [12]. The above studies have shown that *K. mikimotoi* may have contact toxicity characteristics, and its toxicity may be related to intact cells. Hemolytic toxicity is considered to be one of the causes of *K. mikimotoi*-induced fish death. Many studies have demonstrated that *K. mikimotoi* had hemolytic toxicity, which can cause hemolytic effects on different kinds of animal red blood cells, and the toxicity of different algal strains was different [11,12,47]. In this study, *K. mikimotoi* was demonstrated to have a hemolytic effect on rabbit red blood cells, and hemolytic toxicity was positively correlated with cell density. We determined the effects of different densities of intact algal cells, algae-free cell culture and ruptured cell suspension on marine medaka. When the cell density reached 3 × 10^3^ cells/mL, *K. mikimotoi* had rapid lethal effects on marine medaka. Neither algae-free cell culture nor ruptured cell suspension caused the rapid death of marine medaka, and this result is consistent with those of previous studies. MC removed microalgae cells and caused the shape of the algae cells to be irregular or the cells to rupture, thus, the algae cells stuck together, and the edges of the cells were blurred [48]. In this study, MC effectively reduced the cell density of *K. mikimotoi* cells in the water column and affected the external morphology of the algae cells, causing the algae cells to rupture (Figure 1). MC reduced the possibility of marine medaka directly contacting intact algal cells and reduced hemolytic toxicity. Therefore, the toxic effect of *K. mikimotoi* on marine medaka fish was reduced, and the fish survival rate was improved.

### 4.2. The External Morphology and Osmotic Adjustment of Gills

The scanning electron microscopy images in this study (Figure 3) showed that compared with the SW group, many algae cells attached to the gill surface of KM group, and accompanied by mucus production. The gill filaments were obviously shrunken, and the rapid death of marine medaka may be caused by gill damage. There was no significant difference between the MC group and the SW group, and the effect of MC on the surface morphology of fish gills was basically negligible. The gills in the KMMC group had only a small amount of clay particles attached, and the surface structure of the gills was not significantly different from that of the SW group. The addition of MC significantly reduced the adhesion of *K. mikimotoi* cells on the surface of the gills, then greatly reduced the impact of *K. mikimotoi* on fish gills and improved the survival rate of fish.

Ion balance maintains the homeostasis of body fluids, allowing cell activities and physiological processes to proceed normally [49]. NKA plays a central role in osmotic regulation and ion balance because it provides energy for the active transport of Na^+^ and K^+^ across cell membranes and affects the transmembrane movement of cations in the gills [50,51]. Research by Li et al. found that *K. mikimotoi* reduced the NKA activity of rotifers and effected its osmotic regulation function, leading to disturbances in ion transport and basolateral ion pumps and shrinking the surface of rotifers [52]. In this study, compared with the SW group, the NKA activity of the gills in the KM group was significantly reduced (Figure 4A), indicating that *K. mikimotoi* affected the osmotic adjustment function of the gills, while MC reduced this adverse effect, and there was no significant difference in NKA activity between the gills of the KMMC group and the SW group. Compared with the SW group, NKA gene expression (atp1a3a, atp1b2b) in the KM group was also significantly reduced, indicating that the impact of *K. mikimotoi* on NKA was based on both gene expression and enzyme synthesis, and the addition of MC significantly reduced these effects. The claudin family is related to the osmotic regulation of bony fish [53,54]. Compared with the SW group, the expression of CLND-related genes (such as claudin-4, claudin-7-A, claudin-7-B, claudin-8, and claudin-3) in the gills of the KM group was significantly different (Figure S2, Table S3). The difference between the SW group and the KMMC group was small, which also showed that *K. mikimotoi* affected the osmotic adjustment function of fish gills, and this effect was alleviated after adding MC.

### 4.3. Oxidative Stress in Fish Gills

Harmful algae can cause oxidative stress in organisms [55,56]. MDA is the main and most-studied product of PUFA peroxidation, which can be used to quantify the level of oxidative stress in organisms [57]. Antioxidant enzymes, such as superoxide dismutase (SOD) and catalase (CAT), are defence systems that inhibit the formation of ROS. SOD catalyses the conversion of active superoxide anions (O_2_^−^·) into hydrogen peroxide (H_2_O_2_), which itself is also an important ROS. H_2_O_2_ is subsequently decomposed by CATs [58]. In this study, *K. mikimotoi* caused oxidative damage to marine medaka, resulting in an increase in MDA content and SOD activity, and the addition of MC reduced the oxidative damage of *K. mikimotoi* to marine medaka. There was no significant difference in SOD enzyme activity or MDA content between the KMMC group and SW group (*p* > 0.05).

Oxidative stress can cause cell damage by directly acting on molecules or activating intracellular signalling pathways or can induce cell apoptosis [59,60,61]. Figure 7 shows that compared with the SW group, the KM group fish gills had inflammation and immune-related pathways (such as cell adhesion molecules, NOD-like receptor signalling pathway, cytokine–cytokine receptor interaction, cellular senescence, tight junction, apoptosis, C-type lectin receptor signalling pathway, cell cycle, Toll-like receptor signalling pathway, etc.) that were all significantly affected. Apoptosis-related genes, such as TNFα, CHOP, Bax, CASP17, IκB-α, TRAILR2, c-jun, AP-1, Gadd45aa, Gadd45ba, Gadd45ga, and other genes, were significantly up-regulated (Figure S3). Cytokines such as interleukins (ILs) and tumour necrosis factors (TNFs) are markers of inflammation [62,63]. Compared with the SW group, TNFα, IL-1β, IL-11, and other genes were significantly up-regulated in fish gills in the KM group (Figure S3). The changes in the expression of these genes may explain the surface morphological damage caused by *K. mikimotoi* to fish gills to a certain extent. Compared with the SW group, the KMMC group fish gills showed no significant difference in the expression of these genes, indicating that the inflammation and immune response of fish gills were relieved after adding MC.

Combining the morphology, physiology, biochemistry, and transcriptome results, it was found that the effects of *K. mikimotoi* on marine medaka mainly included the following aspects. Intact *K. mikimotoi* that reached a certain density caused marine medaka to rapidly die. This study found that *K. mikimotoi* caused serious damage to fish gills, and it mainly manifested as damage to the external morphology of fish gills, impaired osmotic regulation, oxidative stress induction, inflammation and immune response, and apoptosis. MC is an eco-friendly material and has little impact on marine medaka. The main pathways by which MC has a significant effect on gene expression in gills are cellular senescence, endocytosis, phagosome, and cytokine–cytokine receptor interaction, but the number of enriched genes was small (Figure 7). The addition of MC may have stimulated adaptive defence and repair processes in marine medaka, but it had no significant effect on its main metabolic processes. In addition, MC can significantly reduce the toxicity of *K. mikimotoi*, prolong the survival time of marine medaka, and significantly reduce damage to the gills. Compared with the SW group, the KMMC group exhibited no significant differences in external morphology, NKA activity, SOD activity, MDA content, etc. The number of DEGs was significantly reduced, and the degree of influence on pathways related to osmotic regulation, stress, immunity, and inflammation was significantly reduced. The reason that MC reduces the toxicity of *K. mikimotoi* may be that it effectively reduces the number of algae cells in the water column, causing damage to the remaining and flocculated algal cells, thereby reducing the possibility of marine medaka fish contacting intact *K. mikimotoi* cells. MC reduces hemolytic toxicity and alleviates the hemolytic effect of *K. mikimotoi* on fish gills. The results of the study indicate that MC may be used as an effective and safe method for controlling *K. mikimotoi* blooms.

## 5. Conclusions

We used marine medaka as the model organism to study the toxicity changes of *K. mikimotoi* before and after adding MC. The rapid death of marine medaka caused by *K. mikimotoi* may be related to serious damage to the gills. MC can effectively reduce the toxicity of *K. mikimotoi* to marine medaka, prolong the survival time of fish, and reduce external damage to the gills and other stress responses. These results can provide a certain theoretical basis for using MC in the control of *K. mikimotoi* blooms.

## Figures and Tables

**Table 1:** The relationship between hemolytic toxicity and the cell density of *K. mikimotoi*.

Correlations		Algal Cell Density	Hemolytic Toxicity
Algal cell density	Pearson Correlation	1	0.974 **
	Sig.(2-tailed)		0.000
	N	18	18

**. Correlation is significant at 0.01 level (2-tailed).

**Table 2:** The effects of different components of *K. mikimotoi* on the survival of marine medaka.

Time (h)	Intact Algal Cell Density (Cells/mL)				3000 Cells/mL	
	1000	3000	5000	10000	The Cell-Free Culture Supernatant	The Ruptured Cell Suspension
1	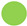	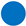	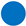	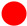	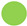	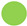
3	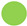	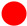	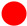	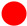	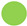	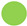
24	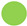	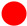	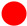	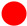	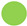	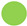
48	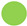	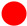	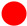	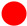	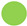	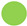
96	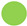	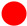	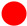	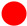	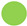	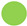

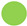
 indicates all were alive, 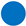 indicates some were alive, 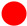 indicates all were dead.

## Data Availability

Data are available upon request; please contact the contributing authors.

## Supplementary Materials

The following are available online at https://www.mdpi.com/article/10.3390/toxics10030105/s1, Figure S1: Standard working curve of digitonin, Figure S2. Claudin family gene expression differences in each group, Figure S3. DEGs heatmap, Table S1. Primers used in this study, Table S2. RNA-Seq data and qRT-PCR data, Table S3. Table of gene names and NR annotations.
