# Supplementary material for: Assessing the Effect of Modified Clay on the Toxicity of Karenia mikimotoi Using Marine Medaka (Oryzias melastigma) as a Model Organism
Source: Toxics. 2022 Feb 23;10(3):105. doi: 10.3390/toxics10030105 (PMC8949556; doi:10.3390/toxics10030105)
Supplement: Supplementary file 1 [file toxics-10-00105-s001.zip › toxics-1576673-supplementary.pdf]

toxics

# Supplementary Materials: Assessing the Effect of Modified Clay on the Toxicity of *Karenia mikimotoi* Using Marine Medaka (*Oryzias melastigma*) as a Model Organism

**Peipei Zhang, Xiuxian Song, Yue Zhang, Jianan Zhu, Huihui Shen and Zhiming Yu**

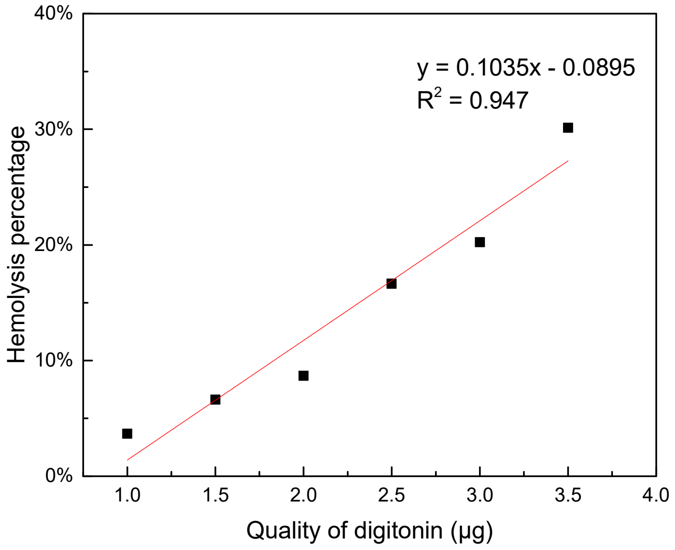


**Figure S1.** Standard working curve of digitonin.

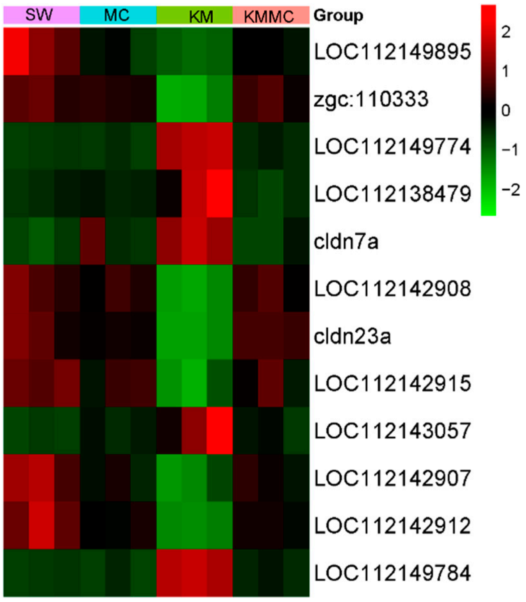


**Figure S2.** Claudin family gene expression differences in each group.

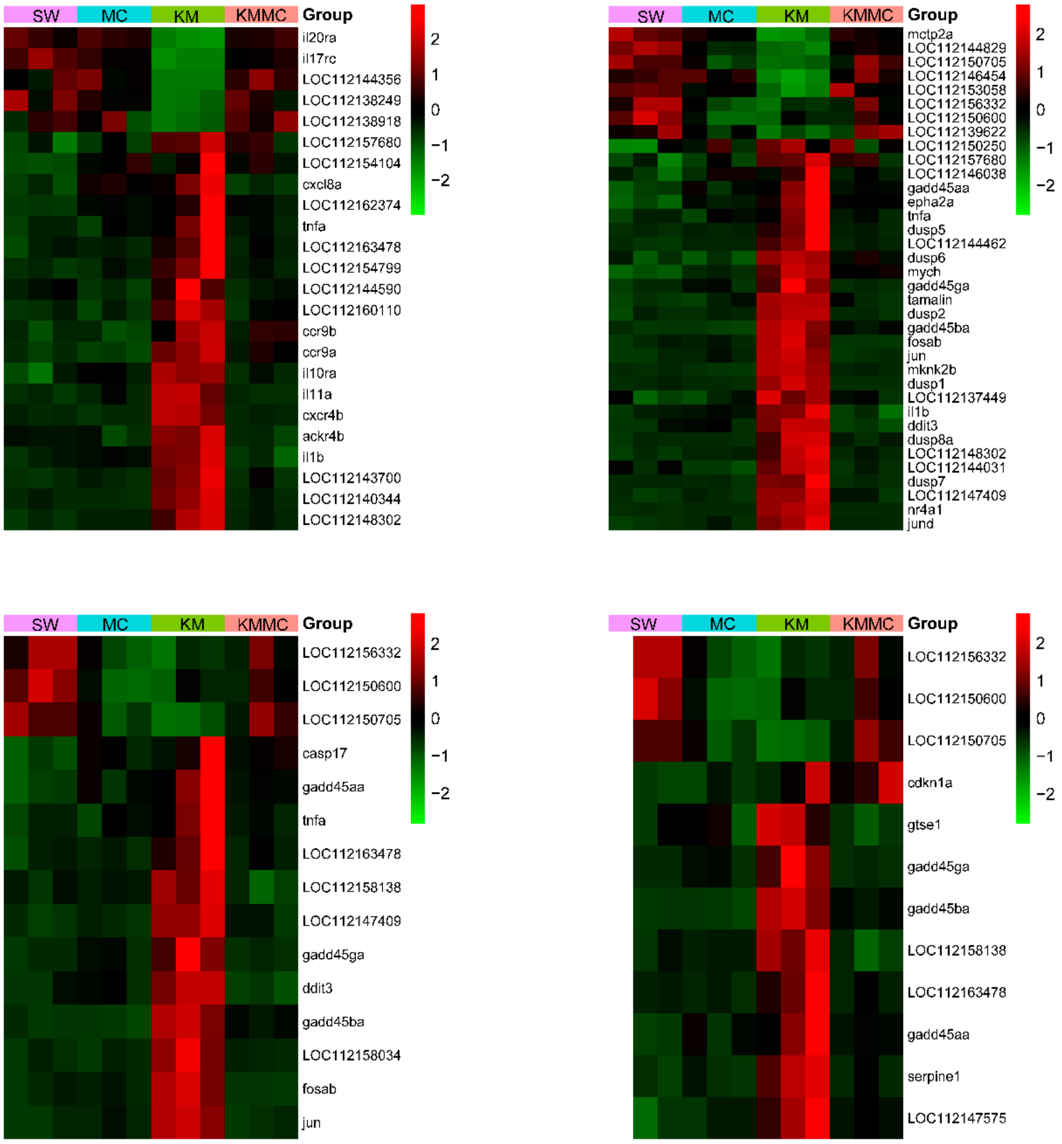


**Figure S3.** DEGs heatmap. Cytokine–cytokine receptor interaction, MAPK signalling pathway, p53 signalling pathway, apoptosis pathway

**Table S1.** Primers used in this study.

| Gene Name | Forward Sequence (5′→3′) | Reverse Sequence (5′→3′) | NR Annotation |
|---|---|---|---|
| *b2m* | AGGAGACCCCAAATGTGTATT | TTTCCTTGTGGTCCAAACTTG | beta-2-microglobulin |
| *TNFa* | CTTATCACGCCCAAAGGTT | GCAGACATTTCCCCATAACT | tumor necrosis factor a |
| *atp1b2b* | TTGCCTGCACATATCCTC | GGGGTTTGAACAGATTTGG | sodium/potassium-transporting ATPase subunit beta-2-like |
| *LOC112148302* | CTGTCTGTCACTGACATGTTTCTG | CAAAGGTGAGACTGTCAAACACAA | interleukin-1 beta |
| *jun* | ACGTTCACCATTAGAGGATTCA | GAAACTCAGGCAGCATTTGT | Jun proto-oncogene, AP-1 transcription factor subunit |
| *il1b* | CCTTCTCAGAGGGTTCAT | GCTTCACTCTGCTCATTAG | interleukin-1 beta |
| *casp17* | TTCTCAGACTGGAACTTTCTCT | CTTTAAGCCGATCCTGATTTGA | caspase17,apoptosis-related cysteine peptidase |
| *LOC112158138* | TGTAGTGTGGGAGAGTTTATCT | AATATCTCAACACTGACTCATCC | apoptosis regulator BAX-like |
| *il11a* | TTAGCCTCTCAGGAAAGTG | GTACAGAAGTGTCGCAATAC | interleukin 11a |
| *ddit3* | GGCTCAAACTGTAATATGCCATCA | GCAGATCCAACGATAAACAGCTT | DNA damage-inducible transcript 3 |
| *LOC112157680* | AATCCTGAAGGAGACTCG | GCTGCTTAGAACTAACCG | transforming growth factor beta-3-proprotein |
| *cxcl8a* | GTGACTATTCAAACAGCCAAT | TTATCGCATGTTCCATCCA | chemokine (C-X-C motif) ligand 8a |
| *cdkn1a* | CACTTCTGCTTATGGTCCTGTA | TCAGATTCATCACGGTTACACA | cyclin-dependent kinase inhibitor 1A |
| *fosab* | CGTTCTCTCTGGTGTTCAA | GGAATGGTCATTGCTGTTG | proto-oncogene c-Fos-like |

**Table S2.** RNA-Seq data and qRT- PCR data.

| Gene Name | log2(FC) qPCR (KM vs. SW) | log2FC) RNA-seq (KM vs. SW) | log2(FC) qPCR (KMMC vs. SW) | log2FC) RNA-seq (KMMC vs. SW) | log2(FC) qPCR (MC vs. SW) | log2FC) RNA-seq (MC vs. SW) |
|---|---|---|---|---|---|---|
| *TNFa* | -0.30632 | 1.42861 | 0.91116 | 0.16809 | -0.31795 | 0.14766 |
| *LOC112158138* | 1.26965 | 1.44716 | -0.60522 | -0.31161 | -0.24855 | 0.04936 |
| *il11a* | 2.20602 | 2.34745 | -0.81764 | 0.09532 | -0.06436 | 0.35987 |
| *ddit3* | -0.26377 | 1.03498 | -0.70158 | -0.15377 | -0.87497 | 0.16288 |
| *gene-LOC112148302* | 6.5833 | 3.06392 | 2.65589 | 0.5467 | 2.92791 | 0.5676 |
| *jun* | 2.61578 | 2.3216 | 0.23787 | 0.12168 | | |
| *fosab* | 2.93203 | 2.42825 | | | 0.46336 | 0.24023 |
| *cdkn1a* | 1.44675 | 1.25684 | | | -0.05184 | 0.27018 |
| *casp17* | | | 4.34989 | 1.06546 | 4.04944 | 0.79705 |
| *LOC112157680* | | 0.75978 | 0.46251 | 0.709 | 0.18014 | |
| *il1b* | 1.03209 | 1.1804 | -0.67874 | -0.26683 | | |
| *cxcl8a* | | | 0.77487 | 0.08362 | | |
| *atp1b2b* | | | 0.00922 | 0.0342 | | |

**Table S3.** Table of gene names and NR annotations .

| Gene Name | NR Annotation |
|---|---|
| *gene-LOC112149895* | claudin-8-like [Oryzias melastigma] |
| *gene-zgc:110333* | claudin-8-like [Oryzias melastigma] |
| *gene-LOC112149774* | claudin-4-like [Oryzias melastigma] |
| *gene-LOC112138479* | claudin-4-like [Oryzias melastigma] |
| *gene-cldn7a* | claudin-7-A [Oryzias melastigma] |

| | |
|---|---|
| *gene-LOC112142908* | claudin-4-like [Oryzias melastigma] |
| *gene-cldn23a* | claudin-23-like [Oryzias melastigma] |
| *gene-LOC112142915* | claudin-3-like [Oryzias melastigma] |
| *gene-LOC112143057* | claudin-7-B-like [Oryzias melastigma] |
| *gene-LOC112142907* | claudin-4-like [Oryzias melastigma] |
| *gene-LOC112142912* | claudin-like protein ZF-A89 [Oryzias melastigma] |
| *gene-LOC112149784* | claudin-4-like [Oryzias melastigma] |
| *gene-LOC112149895* | claudin-8-like [Oryzias melastigma] |
| *gene-zgc:110333* | claudin-8-like [Oryzias melastigma] |
